# Effect of Post-Washing on Textural Characteristics of Carbon Materials Derived from Pineapple Peel Biomass

**DOI:** 10.3390/ma16247529

**Published:** 2023-12-06

**Authors:** Chi-Hung Tsai, Wen-Tien Tsai, Li-An Kuo

**Affiliations:** 1 Department of Resources Engineering, National Cheng Kung University, Tainan 701, Taiwan; ap29fp@gmail.com; 2 Graduate Institute of Bioresources, National Pingtung University of Science and Technology, Pingtung 912, Taiwan; 3 Department of Environmental Science and Engineering, National Pingtung University of Science and Technology, Pingtung 912, Taiwan; sanck112204@gmail.com

**Keywords:** pineapple peel, activated carbon, biochar, post-washing, pore analysis, surface characteristics

## Abstract

Porous carbon materials have been widely used to remove pollutants from the liquid-phase streams. However, their limited pore properties could be a major problem. In this work, the effects of post-washing methods (i.e., water washing and acid washing) on the textural characteristics of the resulting biochar and activated carbon products from pineapple peel biomass were investigated in the carbonization and CO_2_ activation processes. The experiments were set at an elevated temperature (i.e., 800 °C) holding for 30 min. It was found that the enhancement in pore property reached about a 50% increase rate, increasing from 569.56 m^2^/g for the crude activated carbon to the maximal BET surface area of 843.09 m^2^/g for the resulting activated carbon by water washing. The resulting activated carbon materials featured the microporous structures but also were characteristic of the mesoporous solids. By contrast, the enhancement in the increase rate by about 150% was found in the resulting biochar products. However, there seemed to be no significant variations in pore property with post-washing methods. Using the energy dispersive X-ray spectroscopy (EDS) and the Fourier Transform infrared spectroscopy (FTIR) analyses, it showed some oxygen-containing functional groups or complexes, potentially posing the hydrophilic characters on the surface of the resulting carbon materials.

## 1. Introduction

Pineapple (*Ananas comosus*) is a popular and edible fruit for drinking and food industries, but it also generates large amounts of pineapple peel in the industrial and residential sectors. The biomass residue could cause adverse impacts on living quality if dumped into the environment without the proper treatment. Although pineapple peel can be treated by the sanitary landfill and incineration, these treatment methods pose potential drawbacks due to the high contents of moisture and lignocellulose. For example, treatment by thermal combustion at municipal solid waste (MSW) incineration plants not only reduces the energy efficiency for power generation but also could generate huge amounts of air pollutants. In this regard, the traditional options for utilizing pineapple peel are to reuse it as a feedstock for animal feed and a variety of natural products (e.g., organic acids, bioethanol, bromelain enzyme, and phenolic antioxidants) or organic fertilizer by composting [1]. In order to promote the circular economy, the value-added valorization of pineapple peel (PP) has been exploited and reviewed by researchers in recent years [1,2,3,4,5,6]. In the paper by Tran et al. [6], the authors reviewed the production and application of pineapple-derived carbon adsorbents (i.e., biochar and activated carbon), which were produced by thermal processing or chemical modification to improve the surface chemistry and porosity. Other carbon-based nanomaterials (e.g., carbon nanotube and carbon nanosphere) can be also produced from agricultural biomass like rice-based residues [7].

In general, the dried biomass is mainly composed of lignocellulosic constituents, thus containing oxygen-containing groups on the surface. These polar complexes can provide the features of complexation and ion exchange, suggesting that pineapple peel can be directly reused as a biosorbent for removing cationic pollutants from the water environment [6,8,9,10,11,12]. However, these biosorbents only have limited uptake capacities due to their poor pore properties like specific surface area and pore volume. In order to increase the textural characteristics of biomass-derived adsorbent, the biomass precursor must be thermally processed by pyrolysis and pyrolysis-activation methods [13,14], converting it into biochar [15,16,17,18,19,20,21] and activated biochar (activated carbon) [22,23,24,25,26,27,28,29], respectively. The maximal uptake capacities are highly associated with its physical properties like surface porosity and particle size. It has been found that the pore properties of the resulting biochar products derived from pineapple peel indicated relatively low values, which could be attributed to the carbonization conditions at mild temperatures. On the other hand, the production of activated carbon from pineapple peel has almost always been based on chemical activation in the literature [22,23,24,25,26,27,28,29]. It should be noted that the commercial manufacturing process for activated carbon was to employ the physical activation process due to less wastewater treatment problems in comparison with the chemical activation process [1].

In order to enhance the physical and chemical characteristics of biomass-based carbon materials, a plenitude of post-washing treatment methods have been reviewed in the literature [30,31,32]. In a previous study [33], the findings showed that post-washing with deionized water and/or dilute acid can enhance the pore properties of cocoa pod husk (CPH)-derived biochar due to the removal of the inorganic residues and the improvement in the accessibility of the carbon structure by lessoning the pore blockage. For example, the Brunauer–Emmet–Teller (BET) surface area of the resulting biochar produced at 400 °C holding for 30 min was significantly increased from 101 m^2^/g to 342 m^2^/g after post-washing with a dilute acid (0.25 M HCl). The effects of post-washing on the enhancing pore properties of biomass-based carbon materials (i.e., biochar and activated carbon) were also found by other studies [34,35,36].

As mentioned above, it seems that no study has reported on PP-based activated carbon produced by physical activation using carbon dioxide (CO_2_). In order to upgrade the applicability of PP as a precursor for producing carbon adsorbents, the effects of post-washing on the textural characteristics of the resulting carbon materials were investigated in the present study. Based on the results in a previous study [21], the porous carbon products (including biochar and activated carbon) were produced at an elevated pyrolysis/activation temperature (i.e., 800 °C) holding for 30 min in this work because of the significant pore properties increased under the process conditions. Thereafter, the resulting carbon materials were further treated by water washing using deionized water and dilute acid (0.1 M HCl), respectively. The textural characteristics of the resulting carbon products were obtained by the nitrogen (N_2_) adsorption–desorption isotherms for determining pore properties, the scanning electron microscope (SEM) for observing porous structure, and the energy dispersive X-ray spectroscopy (EDS) and Fourier infrared spectrometer (FTIR) for surveying surface elemental compositions.

## 2. Materials and Methods

### 2.1. Materials

The starting feedstock (i.e., pineapple peel) for producing biochar and activated carbon was collected from a local market (Chienchen district, Kaohsiung, Taiwan). It was first dewatered in the sun and further dried in the air-circulating oven at 105 °C. A crusher was used to transform the dried biomass into powdered particles, which were sieved to the size range of 0.841 mm (opening size of mesh No. 20) to 0.420 mm (opening size of mesh No. 40). The dried and sieved biomass sample was used to find its thermochemical characteristics and perform the pyrolysis-activation experiments at 800 °C holding for 30 min.

### 2.2. Thermochemical Property Determination of Pineapple Peel

Due to the different sources, the proximate analysis, calorific value, and thermogravimetric analysis (TGA) of the pineapple biomass were also determined in this work. The adopted methods and analytical instruments refer to a previous study [21]. These thermochemical properties were relevant to the potential for producing carbon materials properly. Herein, the TGA was performed in the temperature range of 25–900 °C at a specific rate of 10 °C/min, which was close to the heating condition of the pyrolysis-activation experiments.

### 2.3. Experimental Methods

As referred to in a previous study [21], the pyrolysis-activation experiments were carried out by using a vertical fixed-bed reactor in this work. For the pyrolysis experiment, about 3 g of the dried pineapple (PP) biomass was used to produce biochar at 800 °C holding for 30 min, where the heating profile was set at about 10 °C/min. For the physical activation experiment, the first stage was to increase the system temperature from 25 °C (room temperature) to 500 °C under the inert atmosphere by purging a nitrogen (N_2_) flow. When the system reached 500 °C, the flowing gas was changed to pass carbon dioxide (CO_2_) by stopping N_2_ gas. Meanwhile, the system was continuously heated to 800 °C at the same rate (about 10 °C/min) holding for 30 min. The resulting biochar (BC) and activated carbon (AC) products derived from PP were further treated by washing with deionized water (WW) and dilute acid of 0.1 M HCl (AW), as based on previous studies [21,37,38]. Therefore, these carbon products were coded as PP-BC, PP-BC-WW, PP-BC-AW, PP-AC, PP-AC-WW, and PP-AC-AW.

### 2.4. Characterization Analysis of Resulting Carbon Materials

As mentioned above, the adopted procedures and analytical instruments for the textural characteristics of the resulting carbon products refer to previous studies [21,39]. It should be noted that the data on specific surface area were based on the Brunauer–Emmett–Teller (BET) model [20,21,22], which were correlated by 4–6 points using the relative pressure (P/P_0_) range of 0.05–0.10. However, the elemental compositions on the surface of the resulting carbon materials were determined by the energy dispersive X-ray spectroscopy (EDS) (model: X-stream-2; Oxford Instruments, Abingdon, UK).

## 3. Results and Discussion

### 3.1. Thermochemical Characteristics of Pineapple Peel (PP)

**Figure 1:**
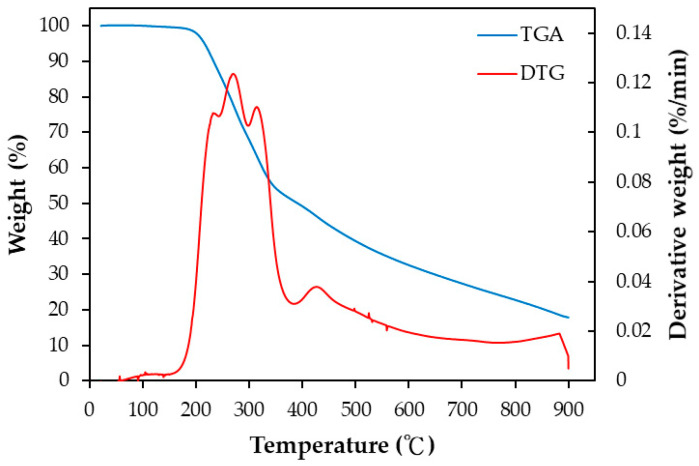
TGA and DTG curves of dried pineapple peel (blue line: TGA; red line: DTG).

According to the American Society for Testing and Materials (ASTM) standards, proximate analysis can give the gross compositions of the biomass without expensive precision instruments. In this work, the dried PP biomass showed the values (in duplicate) of 73.38 ± 2.33 wt% for volatile matter, 4.63 ± 0.25 wt% for ash, and 14.14 wt% for fixed carbon (determined by difference). For this biomass, the ash content had a moderate value, ranging from the low values of woody biomass to the high values of rice residues [40,41]. Using the preliminary analysis of the energy dispersive X-ray spectroscopy (EDS), the elemental contents of the dried PP biomass included carbon (C, 54.07 wt%), oxygen (O, 40.77 wt%), and minor elements, which will be addressed in Section 3.3. Furthermore, the thermogravimetric analysis (TGA) and its derivative thermogravimetry (DTG) curves of the dried PP biomass are shown in Figure 1. The maximal weight loss rate occurred in the pyrolysis temperature range from 200 to 450 °C, which should be attributed to the thermal decomposition of lignocellulosic constituents, especially for hemicellulose [21,22,23]. Based on the TGA results, the physical activation conditions were operated at 500 °C in the first pyrolysis stage to produce a carbon-rich matrix which was subsequently activated at 800 °C by flowing CO_2_ gas.

### 3.2. Pore Analysis of Resulting Carbon Materials

In this work, the process conditions were set at a temperature of 800 °C and a holding time of 30 min using N_2_/CO_2_ gases and post-washing. The mass yields of the resulting carbon materials (i.e., PP-BC and PP-AC) were about 28 wt%. After post-washing with deionized water and dilute acid (i.e., 0.1 M HCl), the residual percentages indicated approximately 84 wt% and 79 wt%, respectively. Concerning the pore properties of the resulting carbon materials (i.e., PP-BC, PP-BC-WW, PP-BC-AW, PP-AC, PP-AC-WW and PP-AC-AW), they are listed in Table 1. Obviously, the values of the resulting biochar products were significantly smaller than those of the resulting activated carbon products. For example, the values of the BET surface area in the PP-BC and PP-AC samples were 100.20 m^2^/g and 569.56 m^2^/g, respectively. The effect of activation by CO_2_ gas on the pore property of the carbon material played a determining role in pore development. On the other hand, the post-washing of the crude carbon products also had an influential effect of enhancing pore property. It could be attributed to the removal of the residual inorganic minerals (or particles) that block the pore entrance, thus leading to more available pores and giving larger pore properties. By comparison, the enhancement in pore property reached about a 50% increase rate, increasing from 569.56 m^2^/g for the PP-AC to 843.09 m^2^/g for the PP-AC-WW or 799.25 m^2^/g for the PP-AC-AW. However, it seemed to show no significant variations on pore property by post-washing methods of DI water and dilute acid. From the viewpoints of economic cost and environmental protection, the post-washing with water was superior to the use of dilute acid in the production of porous carbon materials from pineapple peel.

**Figure 2:**
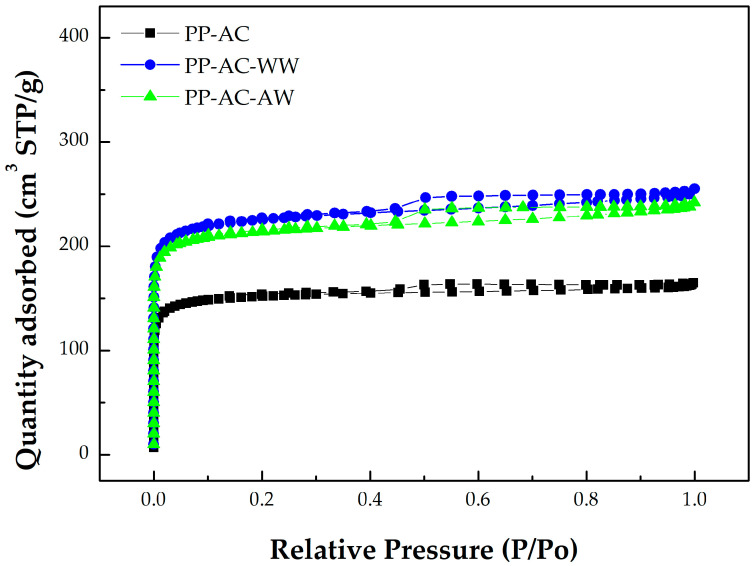
N_2_ adsorption–desorption isotherms of the resulting activated carbon materials.

**Figure 3:**
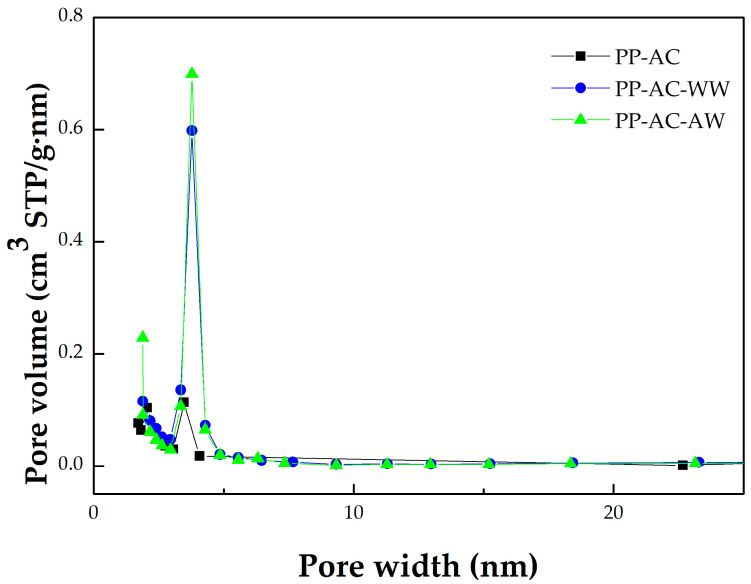
Mesopore size distributions of the resulting activated carbon materials.

**Figure 4:**
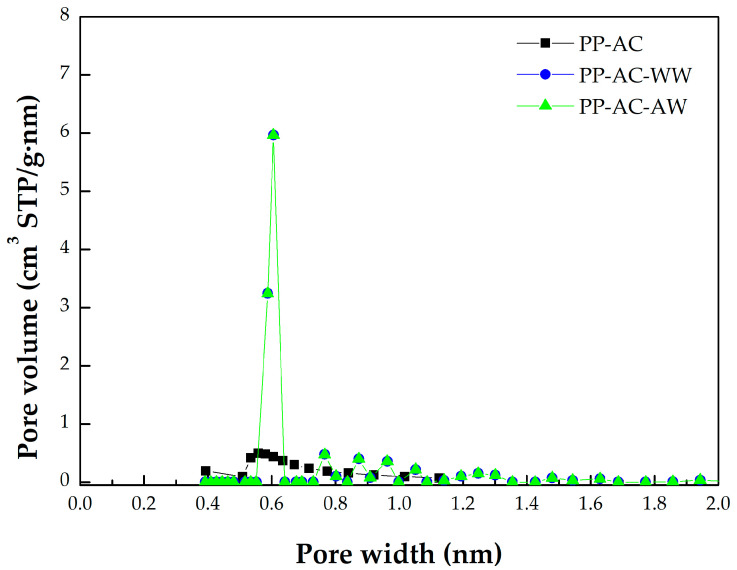
Micropore size distributions of the resulting activated carbon materials.

**Figure 5:**
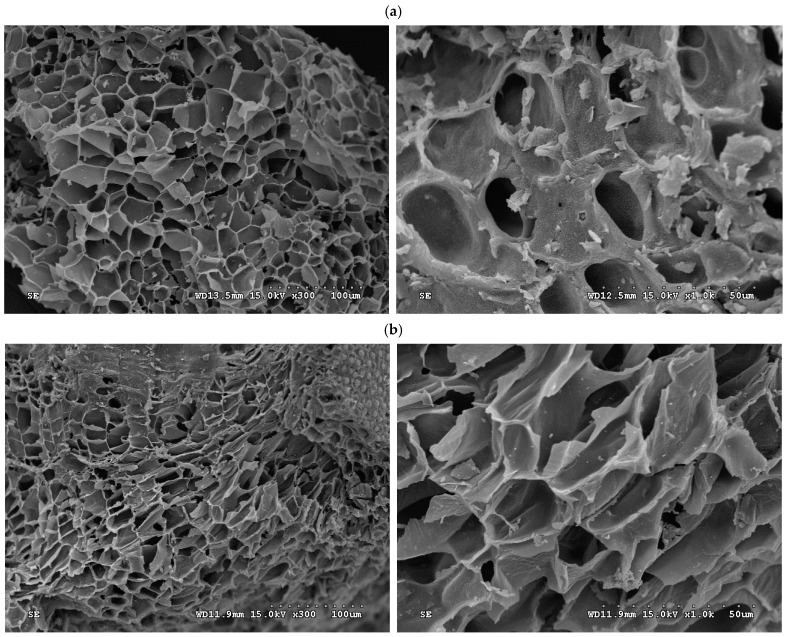

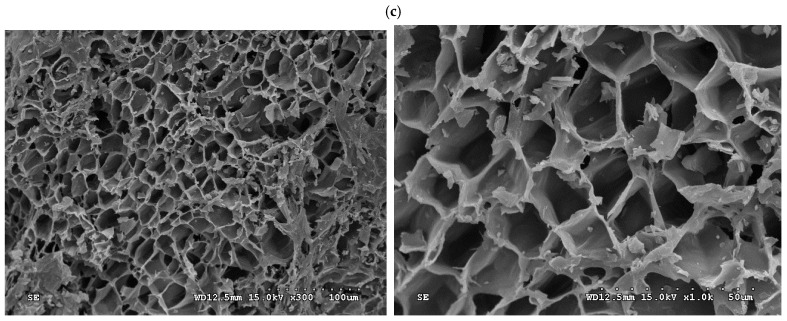
SEM images (left: ×300; right: ×1000) of the resulting activated carbon materials for (**a**) PP-AC, (**b**) PP-AC-WW, and (**c**) PP-AC-AW.

Furthermore, the data in Table 1 can be derived from the N_2_ adsorption/desorption isotherms at −196 °C, mesopore size distributions, and micropore size distributions of the resulting activated carbon products, which are depicted in Figure 2, Figure 3, and Figure 4, respectively. Based on the adsorption–desorption isotherms in Figure 2, the microscale structures of the resulting activated carbon products were mainly microporous. In this regard, the 2D-NLDFT-HS model was adopted to depict their micropore size distributions, which are depicted in Figure 4. These curves were consistent with the data in Table 1. The significant results were further discussed as follows:

1. Based on the classification by the International Union of Pure and Applied Chemistry (IUPAC), these carbon materials are typical of microporous solids, where micropore filling occurs significantly at very low relative pressure (P/P_0_). In this regard, they feature the Type I isotherms [42,43]. However, the adsorption–desorption isotherms also possess a hysteresis loop from the relative pressure of about 0.45, which should be associated with mesoporous solids. This isotherm shape belongs to the Type IV isotherms, where the capillary condensation occurs [42,43].
2. As compared to PP-based activated carbons produced by chemical activation [22,23,25,26,27,28,29], it was clearly shown that the resulting activated carbons produced by physical activation in this work had slightly lower pore properties (e.g., BET surface area), as shown in Table 2. For example, the BET surface area of PP-based activated carbon produced by KOH activation was 1160 m^2^/g [29], which was higher than the optimal value (843 m^2^/g) in Table 2.
3. Using the Harrett–Joyner–Halenda (BJH) method and the data on desorption isotherms for the textural characteristics of the mesoporous solids, it was found that the significant peaks of mesopore size distribution occurred at about 3.5 nm. In addition, these peaks were more obvious for the resulting activated carbon materials (i.e., PP-AC-WW and PP-AC-AW) by post-washing in comparison with the crude product (i.e., PP-AC), thus leading to higher pore properties, as listed in Table 1.
4. As mentioned above, the micropore size distributions of the resulting activated carbon materials can be verified in Figure 3, which was obtained by the 2D-NLDFT-HS model. The micropore peak was observed at about 0.6 nm, where it was in the range of less than 2.0 nm. Assuming the cylindrical geometry for all pores, the data on the average pore diameter (or width) were slightly smaller than 2.0 nm (1.6–1.9 nm). Therefore, other significant peaks in the pore size distribution curves were observed at the left side (less than 2.0 nm), indicating micropores are present in all activated carbon products.
5. In order to see the porous textures on the surface of the resulting carbon materials, Figure 5 shows the scanning electron microscopy (SEM) images (i.e., ×300 and ×1000) for the resulting activated carbon samples (i.e., PP-AC, PP-AC-WW and PP-AC-AW). Obviously, the resulting activated carbon products displayed a porous texture on the rigid surface without significant difference. However, post-washing removed the residual impurities and/or particles, thus producing a cleaner surface and greater pore properties, as listed in Table 1.

### 3.3. Chemical Characteristics of Resulting Carbon Materials

**Figure 6:**
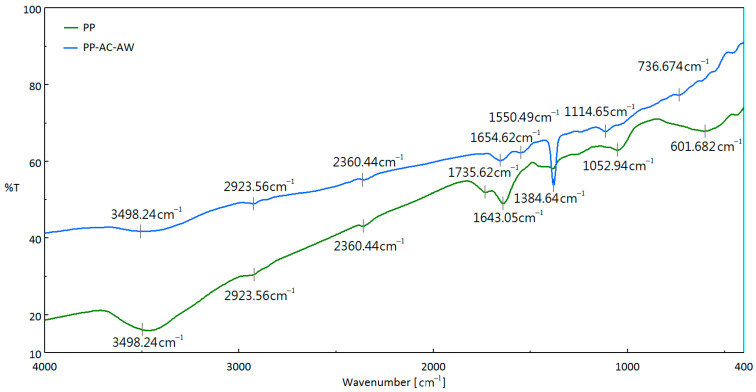
Fourier Transform infrared spectroscopy (FTIR) spectra of pineapple peel (PP) and the resulting activated carbon (PP-AC-AW).

The carbon materials also contain to some extent contents of ash, which is derived from the starting feedstock. These inorganic minerals consist mainly of silica, alumina, alkaline/alkaline earth metals, and transition metals in the forms of oxides and carbonates [44]. On the other hand, these carbon materials are generally hydrophilic because of the presence of oxygen-containing complexes on the surface, which should be derived from their lignocellulosic compositions in the starting feedstock. In order to identify the potential for the polar feature, the resulting carbon products were measured by using the Fourier Transform infrared spectroscopy (FTIR) and the dispersive X-ray spectroscopy (EDS). As seen in Figure 6, it depicts the peaks by % transmittance (T) for the corresponding functional groups of the typical activated carbon (PP-AC-AW) in comparison with the starting biomass (PP). It shows seven peaks in Figure 6, indicating the wavenumbers at around 3500, 2924, 2360, 2655, 1550, 1385, 1115, and 737 cm^−1^, respectively. Based on the FTIR spectra of the corresponding functional groups of carbon materials [45,46,47,48], the peak at around 3500 cm^−1^ generally refers to the hydroxyl (O-H) stretching due to the adsorbed or inherent water molecule (H_2_O). The peak at about 2924 cm^−1^ could be associated with C-H stretching. The peak at 2360 cm^−1^ may be due to the C≡C stretching in alkyne groups. The peaks (i.e., 1655 and 1550 cm^−1^) in the range from 1700 to 1550 cm^−1^ are aligned to C=C stretching. The most significant peak at 1385 cm^−1^ can be attributed to oxygen-containing functional groups like C=O and C–O of carboxylic groups, or O–H bending. The peak at 1115 cm^−1^ could correspond to the stretching vibration of the C–O group. The peak at 737 cm^−1^ may be related to the bending vibration of C-H. It also shows a significant difference between the oxygen-containing functional groups of PP-AC-AW and PP. Due to its highly aromatic structure, the spectrum of the resulting activated carbon seems to not have strong peaks, especially in the hydroxyl (O-H) stretching peak at around 3500 cm^−1^, where it was almost disappeared in the activated carbon spectrum.

Table 2 lists the values of elemental compositions on the surface for all of the resulting carbon materials, which were preliminarily obtained by the energy dispersive X-ray spectroscopy (EDS). Obviously, the carbon contents of the resulting carbon materials (71–89 WT%) were significantly larger than that (54 wt%) of the starting feedstock (i.e., PP), showing that the thermal treatment by carbonization/activation had a determining effect on the textural characteristics. This result was consistent with the variations on the spectra by FTIR analysis. In addition, the carbon contents of the resulting activated carbon materials were smaller than those of the resulting biochar materials. It can be postulated that the oxygen content was reduced because of the reaction of biochar carbon with CO_2_ (gasification gas) and the release of CO gas. On the other hand, the oxygen contents of the resulting carbon materials indicated a ramping reduction. This result can be attributed to the devolatilization of non-carbon elements (e.g., oxygen and hydrogen) from the lignocellulosic constituents in the forms of oxygen-containing gases (i.e., CO, CO_2_, and H_2_O) during the thermochemical process. Concerning the inorganic elements, they could be concentrated on alkaline and alkaline earth metal oxides during the thermal process. These oxygen-containing organic groups and inorganic minerals may increase the hydrophilicity of the resulting PP-based activated carbon materials, which are advantageous for various adsorption treatment processes for effective removal of micropollutants from the aqueous phase. This application can be attributed to the excellent pore structure and electrostatic attraction between the negatively charged surface and cationic targets.

## 4. Conclusions

The effect of post-washing on the textural characteristics of carbon materials derived from pineapple peel biomass was investigated in this study. These carbon products (i.e., biochar and activated carbon) were produced at an elevated temperature (i.e., 800 °C) holding for 30 min under a combined N_2_-pyrolysis and CO_2_-activation process. The findings showed that the effect of activation by CO_2_ gas on the pore property of the carbon material played a determining role in pore development, with the maximal pore properties for the activated carbon product produced by post-water washing (i.e., BET surface area of 843 m^2^/g, and total pore volume of 0.391 cm^3^/g). These resulting carbon materials were porous carbon materials with microporous and mesoporous structures. On the other hand, post-washing of the crude carbon products also had an influential effect of enhancing pore property. However, there seemed to be no significant variations in pore property with post-washing methods of water and dilute acid (0.1 M HCl). According to the results of the energy dispersive X-ray spectroscopy (EDS) and the Fourier Transform infrared spectroscopy (FTIR), the chemical characteristics of the resulting carbon materials were rich in carbon (>70 wt%) and also posed hydrophilicity because of the significant oxygen-containing functional groups or complexes on the surface.

## Figures and Tables

**Table 1:** Pore properties of resulting carbon products.

Pore Property	PP-BC	PP-BC-WW	PP-BC-AW	PP-AC	PP-AC-WW	PP-AC-AW
BET surface area ^a^	100.20	243.60	269.94	569.56	843.09	799.25
*t*-plot micropore area ^b^	92.15	213.14	237.60	498.59	717.90	689.26
External surface area	8.05	30.46	31.34	70.97	125.19	109.99
Total pore volume ^c^	0.042	0.110 ^e^	0.121	0.253	0.391	0.371
*t*-plot micropore area ^b^	0.038	0.089	0.097	0.204	0.294	0.283
Average pore width ^d^	1.661	1.806	1.802	1.778	1.854	1.856

^a^ Based on a relative pressure range of 0.05–0.100 (4–6 points) by the Brunauer–Emmett–Teller (BET) equation. ^b^ Using the *t*-plot method. ^c^ Calculated at a relative pressure of about 0.995. ^d^ Estimated by the ratio of the total pore volume (V_t_) to the BET surface area (S_BET_) (i.e., average pore width = 4 × V_t_/S_BET_). ^e^ *Analogically* estimated by the total pore volume/micropore volume of the PP-BC-AW sample and the micropore volume of the PP-BC-WW sample.

**Table 2:** Elemental contents of resulting carbon products by EDS spectra.

Elemental Content (wt%)	PP	PP-BC	PP-BC-WW	PP-BC-AW	PP-AC	PP-AC-WW	PP-AC-AW
Carbon (C)	54.072	81.826	88.391	85.916	71.120	78.835	74.921
Oxygen (O)	40.773	14.451	10.186	12.402	20.466	15.819	14.406
Sodium (Na)	0.071	0.018	0.007	0.029	0.000	0.000	0.000
Magnesium (Mg)	0.229	0.493	0.075	0.309	0.775	1.132	0.207
Aluminum (Al)	4.470	0.123	0.067	0.150	0.006	0.111	2.555
Silicon (Si)	0.227	0.287	0.254	0.283	1.050	1.371	2.530
Phosphorus (P)	0.101	2.421	0.370	0.131	3.719	1.076	0.409
Sulfur (S)	0.058	0.345	0.544	0.460	1.738	0.632	2.913
Calcium (Ca)	0.000	0.035	0.106	0.319	1.126	1.024	2.060

## Data Availability

Data are contained within the article.
